# The Impact of Antihistamines on Immunotherapy: A Systematic Review

**DOI:** 10.7759/cureus.79421

**Published:** 2025-02-21

**Authors:** Stephanie Nagy, Oksana Denis, Atif Hussein, Marc M Kesselman

**Affiliations:** 1 Rheumatology, Nova Southeastern University Dr. Kiran C. Patel College of Osteopathic Medicine, Davie, USA; 2 Hematology/Oncology, Memorial Cancer Institute, Pembroke Pines, USA

**Keywords:** cationic amphiphilic antihistamines, first-generation antihistamines, histamine receptors, immune checkpoint therapy, immunotherapy, non-cationic amphiphilic antihistamines, second-generation antihistamines

## Abstract

Cancer remains one of the most significant public health challenges globally, contributing to a substantial burden of disease across all populations. Conventional therapies of chemotherapy, radiation, and surgery are commonly used to treat all forms of cancer; however, they all have significant side effects to their use. Immunotherapy has emerged as an effective treatment type for a variety of cancers. As the benefits of immunotherapy in cancer treatment are identified, the interaction between immunotherapy and over-the-counter medication has been explored. Due to the cost and length of time to conduct clinical trials, alternative therapeutics are being examined. Recently, the potential interaction between antihistamines and immunotherapies has gained attention. Six articles were included that analyzed this association. In total 4,171 patients were analyzed with a mean age of 62.66. Cancer types vary between lung (including small-cell and non-small-cell lung cancer), melanoma, hepatobiliary, head and neck, breast, gastrointestinal, renal cell, gynecological, and colon cancers. Among all studies, checkpoint inhibitors were used as a form of immunotherapy. Two studies specifically identified which checkpoint therapies were utilized, including nivolumab, pembrolizumab, ipilimumab, and atezolizumab. All articles found a significant improvement in overall survival rates and longer progression-free rates when antihistamines were added to immunotherapy regimens compared to patients who did not utilize antihistamines. Additionally, some studies also analyzed mortality rates, and each found a significant reduction in mortality rates when antihistamines were paired with immunotherapy. The combination of antihistamines as cancer chemotherapeutics with immunotherapy represents a promising approach to the treatment of cancer. As immunotherapies continue to reshape cancer treatment and as we begin to investigate alternative uses for everyday medications, antihistamines may propose beneficial effects on improving the efficacy of immunotherapy.

## Introduction and background

Cancer continues to be a major global public health challenge, contributing significantly to disease burden across all populations. In 2022, it is estimated that there were nearly 20 million new cases of cancer diagnosed with 9.7 million cancer-related deaths. Nearly 10 million cancer-related deaths have been reported worldwide yearly, with lung cancer being the most common in both incidence and mortality. Following lung cancer, breast cancer, colorectal cancer, and prostate cancer have the greatest incidence, whereas colorectal, liver, and breast cancers, in addition to lung cancer, are the main contributors to cancer-related deaths worldwide [1,2]. Specifically, in the United States, the most prevalent cancers include breast, prostate, lung, colorectal, and melanoma for both sexes of all ages [2]. Cancer remains one of the leading causes of morbidity and mortality globally, necessitating ongoing efforts in prevention, screening, and treatment advancements.

Traditional cancer treatments, including surgery, radiation therapy, and chemotherapy, have long been the cornerstone of cancer management and are widely used in clinical practice [3-5]. Surgery is primarily effective for localized solid tumors; however, it is not suitable for blood cancers like leukemia or cancers that have metastasized, and patients typically require additional treatments following the procedure [6]. In addition to being limited to localized tumors, surgery has a negative impact on healthy tissue and the risk of postoperative complications such as pain, bleeding or clotting disorders, problems with anesthesia, and risk of infection [7]. Radiation therapy can damage nearby healthy cells, leading to various side effects, including oral mucositis, dyspnea, fatigue, hypothyroidism, dysphagia, xerostomia, changes in taste, gastrointestinal (GI) toxicity, sexual dysfunction, fertility concerns, and in very rare cases lung fibrosis [8,9]. Chemotherapy is the conventional method for the treatment of cancers; while effective in targeting cancer cells, it also targets healthy rapidly dividing cells, leading to various dose-dependent side effects such as fatigue, nausea, vomiting, and hair loss, and can compromise the immune system, increasing the risk of infections [3,10]. Additionally, drug resistance is a significant challenge in chemotherapy, characterized by the ability of cancer cells that were initially responsive to anticancer drugs to develop resistance, primarily due to decreased drug uptake and increased efflux mechanisms [4].

The aforementioned side effects of conventional treatments can negatively impact patients' quality of life. Given the significant drawbacks associated with traditional cancer therapies, immunotherapy has recently come to the forefront as a novel therapy method.

Cancer immunotherapy is grounded in the concept that tumors possess immunosuppressive properties, allowing them to evade detection and destruction by the host immune system. Immunotherapy is a systemic therapy generally better tolerated than chemotherapy, as it specifically targets tumor cells, including non-proliferating tumor cells, without harming normal proliferating cells and has fewer side effects [5,11]. Immunotherapy boosts the immune system’s natural ability to detect and destroy cancer cells, leveraging natural mechanisms of self-tolerance and memory to achieve long-term effects [5,11]. It has been approved and shown promising results in treating a variety of cancers, including melanoma, lung cancer, renal cell carcinoma, urothelial carcinoma, head and neck squamous cell carcinoma, hepatocellular carcinoma, specific types of leukemia, and lymphoma [5,12,13]. There are various immunotherapy options available, with immune checkpoint inhibitors (ICIs) being one of the most widely used in cancer treatment. Ipilimumab, a cytotoxic T-lymphocyte-associated protein-4 (CTLA-4) monoclonal antibody, was the first ICI to receive FDA approval in 2011 for treating advanced melanoma [14]. CTLA-4 is a protein receptor found on the surface of T-cells. CTLA-4 inhibitors work by blocking the CTLA-4 receptor on T-cells, which normally acts to suppress immune responses by competing with CD28, another receptor on T-cells, for binding to CD80 and CD86 on antigen-presenting cells (APCs). CTLA-4 binds these ligands with high affinity, sending an inhibitory signal that reduces T-cell activation. By inhibiting CTLA-4, these drugs prevent it from binding CD80 and CD86, allowing CD28 to bind instead. This increased CD28 engagement enhances T-cell activation and proliferation, strengthening the immune response against cancer cells and enabling T-cells to more effectively target cancer cells [13,15]. In addition to CTLA-4, ICIs also target proteins like PD-1 on T-cells and its ligand PD-L1, on APCs, which is often overexpressed on cancer cells [13]. Under normal conditions, PD-1 engagement with PD-L1 dampens T-cell activity to maintain immune tolerance and prevent tissue damage. However, many cancer cells exploit this pathway by expressing high levels of PD-L1, helping them evade immune detection. Blocking PD-1 or PD-L1 disrupts this suppressive interaction, allowing T-cells to remain active and mount a stronger immune response against tumors. Monoclonal antibodies targeting PD-1 or PD-L1 enhance T-cell activation and proliferation, boosting the immune system’s ability to recognize and attack cancer cells [12,13].

As the benefits of immunotherapy in cancer treatment have been identified, the interaction between immunotherapy and over-the-counter medications has begun to be explored; due to the cost and lengthy time to conduct clinical trials, current medications are being examined for alternative therapeutic properties. Recently, the potential interaction between antihistamines and immunotherapies has gained increased attention. Antihistamines traditionally are used in the treatment of allergies and are commonly purchased over the counter. There are two forms of antihistamines, first-generation and second-generation, and both target the H1 receptor; however, due to first-generation antihistamines crossing the blood-brain barrier resulting in drowsiness and somnolence, second-generation antihistamines have grown in popularity [16]. There is potential that first- and second-generation antihistamines may interact in unique ways with immunotherapy due to certain types of cancer cells expressing histamine receptors on their surfaces, and therefore, they can have differing impacts on cancer treatment. Antihistamines can also be classified as cationic amphiphilic antihistamines and non-cationic amphiphilic antihistamines. Cationic amphiphilic antihistamines have a hydrophobic ring structure and a hydrophilic side chain with a cationic amine group, which allows the basic amine group to be protonated when entering the acidic lysosomes leading to a rapid accumulation of the drug, which inhibits lysosomal enzymes resulting in cytotoxicity to cancer cells [17]. Analyzing the relationship that cationic versus non-cationic amphiphilic antihistamines have with immunotherapy is crucial to understanding the potential effectiveness of antihistamines as an adjunctive therapy option (a treatment given in addition to the primary treatment).

This study aims to analyze the impact that antihistamines have on the efficiency of immunotherapy when concurrently used within oncological patients. We hypothesize that the combination of antihistamines with immunotherapy, specifically ICIs in cancer treatment, may boost the antitumor immune response by counteracting the histamine-induced suppression of the immune cells, thereby improving patient outcomes.

## Review

Methods

Search Strategy

A systematic literature review was performed using CINAHL, Ovid (MEDLINE), EBSCO, and Web of Science using the search terms “Immunotherapy OR Checkpoint inhibitors” AND “Cancer” AND “Antihistamines.” Due to the novelty of the field, a secondary Google search was conducted for additional articles using the same search terms; however, no new primary research articles were populated, only ones already identified through traditional databases. This search was done to ensure no novel articles were missed due to the recency of this topic. To ensure the recency of the articles, only articles published between 2010 and 2024 were assessed. The articles were analyzed in a step-wise process by first evaluating the title and abstract for relevance and then assessing the full-text manuscript. The Nova Southeastern University library database was utilized to access databases and full-text articles.

Selection Criteria

**Figure 1:**
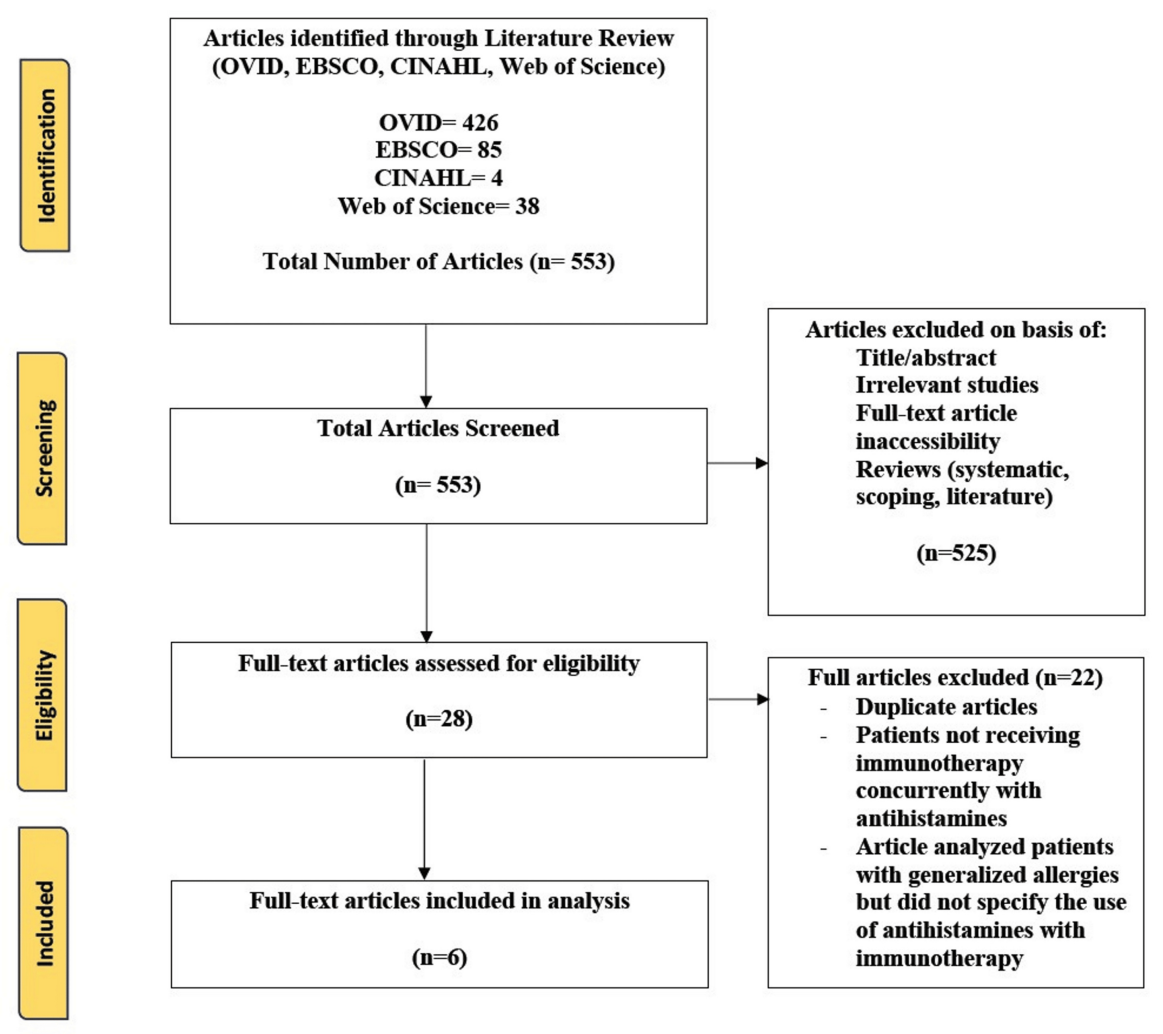
PRISMA diagram indicating the search methodology PRISMA: preferred reporting items for systematic reviews and meta-analyses

For this review, we included randomized controlled trials, cross-sectional studies, observational studies, and cohort prospective/retrospective studies. The population included patients undergoing immunotherapy for cancer treatment, and the intervention was for immunotherapy to be administered in conjunction with a first- or second-generation antihistamine to better understand the impact that antihistamines have on the efficacy of immunotherapy. The outcomes being observed were its impact on the overall survival of patients, progression-free status, and mortality. Studies excluded from this review were literature, systematic or scoping reviews, and animal studies. Articles were excluded if the patient was not receiving immunotherapy for the treatment of their cancer and if patients had a history of allergies but the articles did not provide information on whether antihistamines were used in conjunction with immunotherapy. Two reviewers completed a blinded review process of the articles to decide on their inclusion or exclusion based on the determined criteria, and a third reviewer was used to break any ties. The preferred reporting items for systematic reviews and meta-analyses (PRISMA) guidelines were followed and used to develop a flow diagram of the selection criteria for reproducibility (Figure 1) [18].

The Joanna Briggs critical appraisal tool for retrospective cohort studies was used to assess the risk of bias within the studies analyzed (Table 1).

**Table 1:** Joanna Briggs critical appraisal tool for retrospective cohort studies

Reference	Were the two groups similar and recruited from the same population?	Were the exposures measured similarly to assign people to both exposed and unexposed groups?	Was the exposure measured in a valid and reliable way?	Were confounding factors identified?	Were strategies to deal with confounding factors stated?	Were the groups/participants free of the outcome at the start of the study (or at the moment of exposure)?	Were the outcomes measured in a valid and reliable way?	Was the follow-up time reported and sufficient to be long enough for outcomes to occur?	Was follow-up complete, and, if not, were the reasons for the lost to follow-up described and explored?	Were strategies to address incomplete follow-up utilized?	Was appropriate statistical analysis used?	Overall appraisal
Chiang et al. [19]	Yes	Yes	Yes	No	No	Yes	Yes	Yes	Yes	NA	Yes	Include
Chiang et al. [20]	Yes	Yes	Yes	Yes	Yes	Yes	Yes	Yes	Yes	NA	Yes	Include
Eylemer Mocan et al. [21]	Yes	Yes	Yes	Yes	Yes	Yes	Yes	Yes	Yes	NA	Yes	Include
Li et al. [22]	Yes	Yes	Yes	Yes	Yes	Yes	Yes	Yes	Yes	NA	Yes	Include
Zhang et al. [23]	Yes	Yes	Yes	Yes	Yes	Yes	Yes	Yes	Yes	NA	Yes	Include
Mallardo et al. [24]	Yes	Yes	Yes	Yes	Yes	Yes	Yes	Yes	Yes	NA	Yes	Include

Results

In total, 553 articles were populated between the four databases (OVID, EBSCO, Web of Science, and CINAHL). After the first level of screening, 525 articles were removed based on title, abstract, full-text availability, any form of reviews, publication year, and English language availability, with 28 articles eligible for the second round of screening, in which full texts were completely screened. Articles were removed if they examined patients using forms of treatment other than immunotherapy, if antihistamines were not used concurrently with immunotherapy for cancer therapy, and if patients with allergies were examined but the specification was not provided on the type of antihistamine used and if they were used concurrently with immunotherapy. Six articles were included in the final review.

Table 2 depicts the studies analyzed, including the number of patients, average age, type of cancer diagnosis, type of immunotherapy received, type of antihistamine received, classification of the first or second generation of antihistamine, and the outcomes, including overall survival, progression-free state, mortality, and any additional information presented.

**Table 2:** Analysis of the number of patients, mean age of patients, cancer type, immunotherapy type, antihistamine type and the classification as first- or second-generation, and findings of the articles including the impact on overall survival, progression-free state, and mortality

Title	Number of patients receiving antihistamines	Age of patients (years)	Cancer type	Immunotherapy received	Antihistamine received	Classification of first- versus second-generation antihistamine	Overall survival (OS)	Progression-free state (PFS)	Mortality	Additional findings
Chiang et al., 2022 [19]	68	62	Lung (n = 38), hepatobiliary (n = 16), gastrointestinal (n = 2), head and neck (n = 5), breast (n = 2), renal (n = 3), gynecological (n = 1), other subtype (n = 1)	Not specified, only states immune checkpoint inhibitors	Desloratadine, cyproheptadine, ebastine	Cyproheptadine (first-generation), desloratadine (second-generation), ebastine (second-generation)	Greater overall survival in patients receiving antihistamines (p < 0.018) of 24.8 months versus 10.4 months	Greater progression-free survival rate in patients receiving antihistamines of 16.8 months versus 4.9 months (p < 0.004); lower risk for disease progression (p < 0.002)	All-cause mortality reduced by 50% (p < 0.020)	Higher doses of antihistamines had greater clinical outcomes than lower doses (85% versus 68%) (p < 0.086)
Chiang et al., 2023 [20]	294	Not reported	Lung cancer (n = 294)	Not specified, only states immune checkpoint inhibitors	Not specified; only classified as first- and second-generation antihistamines	Not specified	Longer overall survival in patients receiving antihistamines of 24.4 months versus 6.4 months (p < 0.002); cationic amphiphilic antihistamine had a longer overall survival of 28 months versus 17.8 months (p < 0.015) in non-cationic amphiphilic antihistamine users	Longer progression-free survival in patients receiving antihistamines of 8.2 months versus 4.1 months (p < 0.049)	All-cause mortality and disease progression reduced by 35%-50%	Not mentioned
Eylemer Mocan et al., 2023 [21]	55	62	Melanoma (n = 20), renal cell carcinoma (n = 9), non-small-cell lung carcinoma (n = 13), small-cell lung cancer (n = 10), other (n = 3)	Nivolumab (n = 28), pembrolizumab (n = 7), ipilimumab (n = 9), atezolizumab (n = 11)	Pheniramine (n = 47), cetirizine (n = 4), desloratadine (n = 3), fexofenadine (n = 1)	Pheniramine (first-generation), cetirizine (second-generation), desloratadine (second-generation), fexofenadine (second-generation)	Longer overall survival in patients receiving antihistamines of 16.2 months versus 7.7 months (p < 0.002)	Longer progression-free survival in patients receiving antihistamines of 8.2 months versus 5.1 months (p < 0.016)	Not reported	Rates of adverse events were unchanged at 10.9% versus 7.7% (p < 0.552)
Li et al., 2022 [22]	3,544	Not reported	Melanoma (n = 878), lung (n = 1,937), breast (n = 342), colon (n = 387)	Anti-PD-1/PD-L1	Not specified; only classified as first- and second-generation antihistamines	Not reported	Significant greater overall survival in patients receiving antihistamines (p < 0.0016)	Significant greater progression-free survival in patients receiving antihistamines (p < 0.0023)	Reduced death rates	Adverse events were similar at 10.9% in antihistamine users versus 7.7% in non-users (p < 0.552)
Zhang et al., 2024 [23]	139	Not reported	Lung cancer (n = 139)	Immune checkpoint inhibitors	Diphenhydramine (n = 40), diphenhydramine with cimetidine (n = 69), cimetidine (n = 27)	Diphenylamine (first-generation), cimetidine (second-generation)	Significant greater overall survival in patients receiving antihistamines of 32.8 months to 18.1 months (p < 0.038)	Significant greater progression-free survival in patients receiving antihistamines of 12.7 months to 4.3 months (p < 0.001)	Not reported	Diphenhydramine on its own had longer overall survival and progression-free survival compared to diphenhydramine and cimetidine together (PFS p < 0.001, OS p = 0.030); only cimetidine had lower progression-free survival (5.8 months versus 4.1 months, p < 0.001) and overall survival (25.5 months versus 16.9 months, p = 0.047) than the control group; H1 antihistamines had a good prognostic factor; H2 antihistamines had a poor prognostic factor
Mallardo et al., 2022 [24]	71	63	Melanoma (n = 71)	Pembrolizumab (n = 25), nivolumab (n = 46)	Cetirizine	Cetirizine (second-generation)	Significant greater overall survival in patients receiving antihistamines of 36 months compared to 23 months (p < 0.0032)	Significant greater progression-free survival in patients receiving antihistamines of 28 months to 15 months (p < 0.0023)	Not reported	Elevation of Fc receptor I (FCGR1A/CD64), C–C motif chemokine 8 (CCL8), interferon-induced antiviral RNA-binding protein (IFIT1), IFN-induced antiviral protein (IFIT3), and interferon-inducible antiviral protein (RSAD2)

**Figure 2:**
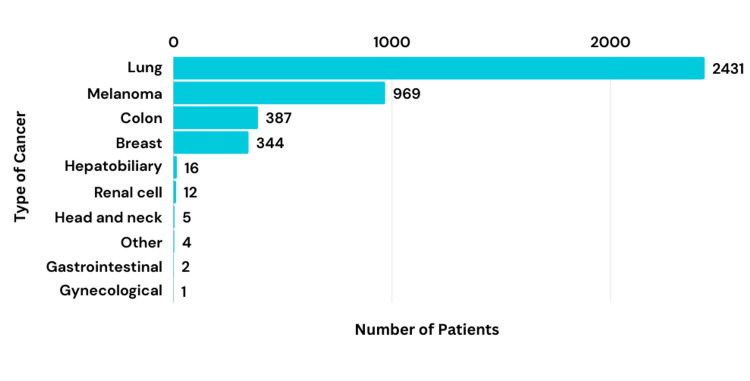
Type of cancer diagnoses among the patients analyzed

In total, 4,171 patients were analyzed. The mean age of patients was reported in half of the studies, with a mean age of 62.66. Cancer types vary between lung (including small-cell and non-small-cell lung cancer) (n = 2,431), melanoma (n = 969), hepatobiliary (n = 16), head and neck (n = 5), breast (n = 344), GI (n = 2), renal cell (n = 12), gynecological (n = 1), and colon (n = 387) cancers, and two studies group the remaining types of cancers as “other” (n = 4) but did not specify those specific types (Figure 2).

Among all studies, checkpoint inhibitors were used as the form of immunotherapy. Two studies specifically identified which checkpoint therapies were utilized including nivolumab, pembrolizumab, ipilimumab, and atezolizumab. The most common is nivolumab, followed by pembrolizumab. Four of the six studies identified which antihistamines were used by patients, with two studies lacking specification. The first-generation antihistamines included were cyproheptadine, pheniramine, and diphenylamine, and the second-generation antihistamines included were desloratadine, ebastine, cetirizine, desloratadine, fexofenadine, and cimetidine. When analyzing the findings of the articles, all articles found a significant improvement in overall survival rates and longer progression-free rates when antihistamines were added to immunotherapy regimens compared to patients who did not utilize antihistamines. Additionally, some studies also analyzed mortality rates, and each found a significant reduction in mortality rates when antihistamines were paired with immunotherapy [19,20,22]. Interestingly, Chiang et al. found that specifically cationic amphiphilic antihistamines improved outcomes to a greater extent than non-cationic amphiphilic antihistamines [20]. Furthermore, Zhang et al. compared first- and second-generation antihistamines and found that first-generation improved survival rates (p < 0.038) and progression-free status (p < 0.001) to a greater extent than second-generation antihistamines [23].

Discussion

Immunotherapy drugs have been approved for a variety of therapies for immunological diseases to malignancies. Immunotherapy has revolutionized the field of oncology. Immunotherapies work to upregulate or downregulate to modulate the patient’s immune system to target their specific condition’s needs [25]. The use of immunotherapies as cancer treatments has been an evolving field with the utilization of the patient’s own immune system against cancer cells. Currently, there are 11 ICIs, six CAR-T therapies, and six T-cell-enhancing antibodies approved for the treatment of various cancers [26]. ICIs are currently the most widely used with approximately 40% of patients with cancer meeting eligibility to initiate this therapy for their oncological treatment; they also cover the widest array of cancers, the most common being non-small-cell lung carcinoma, small-cell lung carcinoma, and hepatocellular carcinoma [27].

Antihistamines are commonly used for allergic reactions to target the H1 histamine receptors to reduce allergy symptoms [28]. There are antihistamines that bind to H2 histamine receptors; however, those target the GI system. There are two forms of antihistamines: first- and second-generation. First-generation is less utilized because it crosses the blood-brain barrier, leading to sedation and anticholinergic effects, while second-generation is preferred because it remains within the peripheral nervous system and has less sedating effects [28]. Outside of the conventional treatment of allergies, antihistamines are also used for sinusitis, motion sickness, bronchitis, nausea, vomiting, peptic ulcer disease, gastritis, gastroesophageal reflux disease, and Zollinger-Ellison syndrome [28]. Due to the diversity of antihistamine use and the costly, timely, and resource-intensive process to develop new oncological therapy, antihistamines have begun to be examined for their use in augmenting immunotherapy.

With cancer being the leading cause of mortality, the development of adjunctive therapy options to improve the efficacy of current treatments is critical. The reason behind the selection of antihistamines for further evaluation to augment immunotherapy is the presence of histamine receptors on cancer cells. There are four histamine receptors: H1, H2, H3, and H4. These histamine receptors have been found to modulate cell proliferation, invasion, inhibition of apoptosis, migration, and vascularization [29-31]. High histamine content has been found in different human tumors including melanoma, colon, and breast cancer. Also, it was found that cancers that had H1, H2, H3, and H4 receptors induced a favorable cancer microenvironment [32]. Furthermore, specifically, the upregulation of H1 and H2 receptors in cancer cells has been associated with worse prognoses for patients [29,31,33,34]. As a result, the investigation into the use of antihistamines that target these receptors is critical to inhibit cancer progression and improve the overall survival of patients.

Antihistamines come in two forms, first-generation and second-generation, that differ in their ability to cross the blood-brain barrier. A variety of each kind was analyzed within the studies. First-generation antihistamines used included cyproheptadine, pheniramine, and diphenylamine. Second-generation antihistamines used included desloratadine, ebastine, cetirizine, desloratadine, fexofenadine, and cimetidine. The majority of studies did not analyze the effectiveness of first- and second-generation antihistamines, even when patients were divided on the medication they consumed. Only one study [23] analyzed this difference and found that those who used diphenhydramine, a first-generation antihistamine, had longer overall survival (p < 0.038) and progression-free survival compared to cimetidine, a second-generation antihistamine. However, in the literature, studies have found that there is greater (p < 0.001) evidence of second-generation antihistamines improving outcomes and survival but little evidence of first-generation antihistamines [35]. As a result of the conflicting findings, further research is required to better understand if there is a benefit to the use of first- or second-generation immunotherapy in improving the efficacy of immunotherapy or if both are equally effective.

Furthermore, antihistamines are divided into cationic and non-cationic amphiphilic antihistamines. Cationic antihistamines have been found to have greater anticancer effects when compared to non-cationic amphiphilic antihistamines. Cationic amphiphilic antihistamines are defined by their hydrophobic ring structure and hydrophilic side chain with a cationic amine group. When placed in an acidic environment, such as a lysosome, the basic amine group gets pronated and leads to a significant accumulation. When cationic antihistamines enter the lysosome, they inhibit lysosomal lipases by neutralizing the negative charge; specifically, they inhibit sphingomyelinase leading to sphingomyelin accumulation, which is toxic to cancer cells [36-39]. Cationic antihistamines, including desloratadine, cyproheptadine, ebastine, loratadine, and astemizole, have been found to be associated with greater overall survival and longer progression-free survival when compared to non-cationic amphiphilic antihistamines [20,40]. Additionally, cationic antihistamines have been shown to have greater anticancer properties through modulating genes. They have recently been found to accumulate inside lysosomes due to their unique structure to rapidly increase the lysosomal pH, which elevates the efflux of hydrogen, leading to cancer cell apoptosis. It also enhances the inhibition of the STAT3 gene, causing tumor growth restriction [41]. It would be beneficial to further analyze the use of cationic antihistamines with immunotherapy in cancer therapy to better understand its impact on patient outcomes.

With the common use of antihistamines in the treatment of allergies, these patients may already be at an advantage in preventing cancer. It has been found that tissues in the body that are linked to different forms of allergies, including the skin and GI tract, were found to have lower rates of cancer within these tissues. Also, most studies showed an inverse relationship between allergies and cancer development [42]. However, the data out there is conflicting as one study found that those with pre-existing allergies had a lower risk of developing glioma, colorectal cancer, cancer of the larynx, non-Hodgkin lymphoma (a cancer of the esophagus), oral cancer, pancreatic cancer, stomach cancer, and uterine body cancer, but an increased risk for bladder cancer, lymphoma, myeloma, and prostate cancer [43]. It specifically was found that those with allergies had lower risks of cancers within tissues and organs that interact with the external environment versus higher risks in the tissues and organs that do not interact [42]. The debate continues to understand the association between allergies and the development of cancers later on; potentially, it could be hypothesized the amount of antihistamines patients consume for their allergies may be a protective factor against the development of cancer, but further research is required.

This review is subject to several limitations that should be considered when interpreting the findings. First, the number of studies included in the analysis was relatively small due to the novelty of the field, which may limit the generalizability and robustness of the conclusions drawn. Additionally, many of the studies did not provide detailed information regarding the duration or dosage of the antihistamines used, making it difficult to assess and compare the full impact of treatment regimens on outcomes. Another significant limitation is the lack of clarity regarding the specific types of immunotherapy employed in the included studies. Further research is required to understand the effectiveness of antihistamines with different forms of immunotherapy. Moreover, there remain open questions regarding the comparative effectiveness of first- versus second-generation antihistamines, as well as the distinction between cationic and non-cationic amphiphilic antihistamines. Only one study compared cationic to non-cationic amphiphilic antihistamines, and only one compared first- to second-generation antihistamines [20,23]. In conclusion, while this review provides valuable insights, further investigation with larger, more detailed studies is essential to address these limitations and provide more definitive conclusions.

## Conclusions

The combination of antihistamines with immunotherapy represents a promising approach to the treatment of cancer. As immunotherapies continue to reshape cancer treatment and as we begin to investigate alternative uses for everyday medications, antihistamines may propose beneficial effects on improving the efficacy of immunotherapy. Novel findings all indicate the positive effects immunotherapy in combination with antihistamines have on overall survival and progression-free survival states. While more research is needed to confirm these benefits and understand the underlying mechanisms, this combination approach holds considerable promise for improving patient outcomes and advancing the way we treat cancer.
